# Three-Year Custody Outcomes Among Infants Investigated by Child Protection Systems for Prenatal Substance Exposure in California

**DOI:** 10.1007/s10995-023-03690-9

**Published:** 2023-05-31

**Authors:** Julia Reddy, Lindsey Palmer, Emily Putnam-Hornstein

**Affiliations:** 1 Gillings School of Global Public Health, University of North Carolina, 412 Rosenau Hall, Chapel Hill, NC 27599 USA; 2 The Pennsylvania State University, 133 Health and Human Development Building, University Park, PA 16802 USA; 3 School of Social Work, University of North Carolina at Chapel Hill, Tate-Turner-Kuralt Building, 325 Pittsboro St, Chapel Hill, NC 27599-3550 USA

**Keywords:** Prenatal substance use, Child protection system, Custody, Foster care, Adoption, SUD

## Abstract

**Objective:**

Infants affected by prenatal alcohol and drug use are more likely to be removed from parental custody than those in the general population, although it is unclear whether their custody outcomes differ from infants investigated by child protection systems (CPS) for other reasons. This analysis seeks to compare trajectories of involvement and custody outcomes among infants investigated by CPS with and without documentation of prenatal substance exposure (PSE).

**Method:**

We used vital birth records linked to administrative CPS records to examine the timing of system involvement and 3-year custodial outcomes among investigated infants with and without identified PSE. We defined PSE according to documentation on the state’s standardized hotline screening form, which CPS completes upon referral for alleged maltreatment. We estimated the likelihood a child was in nonparental custody at age 3 by specifying multivariable generalized linear models, adjusted for covariates available in the birth record.

**Results:**

In our sample of 22,855 infants investigated by CPS in 2017 in California, more than 26% had documentation of PSE. These infants experienced an accelerated timeline of system penetration and were 2.2 times as likely to be in nonparental placement at age 3.

**Discussion:**

PSE confers an independent risk of custody interruption among infants investigated by CPS. The younger age of these infants, complexity of parental substance use, and potential misalignment of administrative permanency timelines with parental recovery all suggest the need for increased research, policy, and programmatic interventions to serve this vulnerable population.

## Significance

Children with PSE face environmental risks in the early developmental period. Often in the United States, CPS is relied on to assess and mitigate these risks. Amid calls for a public health response to PSE, it is essential to understand how children with PSE interact with CPS. We describe the incidence and timing of custody interruptions in a large U.S. state, comparing infants with PSE to those investigated by CPS for other reasons. This study extends current understanding by demonstrating the independent risk of custody interruption conferred by PSE status.

Drug and alcohol use among women of reproductive age has increased in recent years (Center for Behavioral Health Statistics and Quality, 2020), including during pregnancy (Chang, 2020). In 2019, estimates suggest that more than 12% of pregnancies in the United States were exposed to drug or alcohol use (Center for Behavioral Health Statistics and Quality, 2020). Nationally, rising prenatal substance use has been associated with increasing rates of child maltreatment reporting and foster care placements (Ghertner et al. 2018; Lynch et al. 2018; Meinhofer & Angleró-Díaz 2019; Radel et al.  2018). In 2019, 47 states cumulatively reported more than 38,000 child maltreatment referrals related specifically to prenatal substance exposure (PSE), of which more than 32,000 were screened in by child protection systems (CPS) for investigation or alternative response (Children’s Bureau, 2021). Although this represents an increase from the almost 28,000 infants reported by 45 states in 2018 (Children’s Bureau, 2020), these are likely underestimates of infants affected by PSE in the United States, given variability in PSE identification and child maltreatment reporting policies and practices (Coleman-Cowger et al. 2019; Paris et al., 2020; Rebbe et al. 2019).

Despite calls for a public health response to PSE (Patrick et al., 2017; Terplan et al., 2015), CPS remains the primary agency called on to investigate and intervene when PSE is identified. Some states in the U.S. require a maltreatment report to be filed at the time of birth for any identified PSE (universal reporting), including prescription medications used appropriately to treat addiction (Lloyd et al., 2019). Other jurisdictions, such as California, require the identification of additional risk factors before a hospital provider files a report of suspected maltreatment regarding a substance-exposed birth (California Penal Code, 2000). Even in the absence of universal reporting policies, most PSE-identified infants are reported to CPS. In California, more than 50% of a cohort of infants with PSE identified at birth were reported to CPS in the first month of life (Putnam-Hornstein et al., 2016). Infants with PSE are 5 times as likely to be reported to CPS and more than 10 times as likely to be placed in foster care in their first year compared to infants without identified exposure (Prindle et al., 2018).

Although it is well documented that PSE-identified infants have heightened rates of CPS interactions, very little is known about the patterns of CPS involvement and foster care placement for PSE-identified infants compared to infants reported to CPS for other reasons. In a recent systematic review of 30 studies related to PSE and CPS involvement (Austin et al., 2021), only one study compared outcomes among infants with open CPS cases, finding an increased risk of subsequent CPS reports associated with PSE status (Smith & Testa, 2002). Three studies compared CPS outcomes among children placed in foster care, finding that those with documented PSE were more likely to remain in foster care for longer periods, experienced more placement transitions, and when reunified, were more likely to reenter foster care than their nonexposed counterparts (Frame, 2002; Lewis et al., 1997; Smith et al., 2007).

PSE often co-occurs with other familial risk factors, including parental mental health disorders (Benningfield et al., 2010; Racine et al., 2021), limited financial resources (Lloyd, 2018; Stritzel, 2022), and relational instability or violence (Pallatino et al., 2021; Velez et al., 2006). For these reasons, comparisons between infants affected by PSE and a general population or birth cohort may mask intersecting vulnerabilities of the exposed population (Ettekal et al., 2020; Weber et al., 2021). Because PSE is inconsistently screened, disclosed, documented, and reported, delineations between infants with and without PSE are imperfect (Ellsworth et al., 2010; Paris et al., 2020). It is not well known how many infant maltreatment investigations involve PSE (Austin et al., 2021). Additionally, few studies have been able to link population-level birth or medical records to CPS data, meaning we still don’t fully understand whether PSE-identified infants move through CPS in a unique way as a group (Crowley et al., 2019).

The current study explored whether custodial outcomes—meaning a foster care, adoptive, or guardianship placement, as opposed to the parental home—differ among PSE-identified infants compared to infants investigated by CPS for other reasons. Using linked administrative records from California, we identified a population-based cohort of infants born in 2017 who were investigated by CPS within the first year of life. We followed these infants through age 3 years to examine patterns of system involvement and custodial outcomes according to whether the first investigated CPS report relating to that child contained documented PSE. Our primary objective was to estimate the likelihood of being in nonparental custody at age 3 by PSE status, both with and without adjustments for other demographic factors.

## Method

### Study Cohort

We used vital statistical birth records from the California Department of Public Health probabilistically linked to administrative CPS data from the California Department of Social Services. Records were matched using a combination of nonunique (e.g., first name, date of birth) parental and child identifiers. Details of the linkage methodology have been described in prior publications (Putnam-Hornstein et al., 2020).

The current analysis included all children born in California between January 1 and December 31, 2017, who were the subject of an investigated CPS report of abuse or neglect in their first year (*N* = 23,279). Our population of children investigated for maltreatment represents 4.9% of the 2017 California birth cohort, a share consistent with similar analyses (Children’s Bureau, 2019; Putnam-Hornstein et al., 2015). Because our study focused on custody interruptions, which are conditional on CPS investigation, we restricted our analytic sample to infants who were the subject of at least one report screened in for investigation.

### Exposure Variable

The exposure of interest (PSE) was measured according to whether PSE was documented on the state’s standardized hotline screening form, which CPS completes upon referral for alleged maltreatment (Cuccaro-Alamin et al., 2017). The screener specifies whether “there is a positive toxicology finding for a newborn infant or his/her mother OR other credible information that there was prenatal substance abuse by the mother AND there is indication that the mother will continue to use substances, rendering her unable to fulfill the basic needs of the infant upon discharge from the hospital” (California Department of Social Services, 2021), with responses of yes or no. This PSE field was missing for 375 referred infants in our sample (1.6% of the cohort). Those infants were excluded, but sensitivity analyses confirmed how they were coded or handled in our data would not affect our estimates. We also excluded 24 children who were known to have died based on available records and 25 children who had ambiguous dates of contact or foster care termination reasons (< 1% of the cohort). Our final analytic sample included 22,855 infants.

### Outcome Variable

We defined our outcome as whether a child was in nonparental custody at their third birthday. Children who had never been removed by CPS or had been removed but were reunified at age 3 were coded as 0; children in an open foster placement or who had exited foster care to adoption or guardianship were coded as 1.

For our descriptive analyses, we additionally examined children in nonparental custody stratified by open foster care placement or a more permanent transfer of custody via adoption or guardianship. The 3-year study window was chosen because it incorporates the full length of the federally enacted permanency timeline, imposed by the American Safe Families Act (1997), which instructs jurisdictions to hold a permanency hearing within 12 months of removal from the parental home and terminate parental rights if a child spends 15 of the prior 22 months in nonparental care.

### Covariates

Prior literature was used to identify sociodemographic characteristics documented in the birth record that may indicate a heightened risk of alleged or substantiated child maltreatment (Wu et al., 2004), custody interruptions (Putnam-Hornstein & Needell, 2011), and injury and death (Schneiderman et al., 2021; Vaithianathan et al., 2018). Coding of maternal race and ethnicity followed conventions in which primary race is overridden by indication of Hispanic ethnicity. Among mothers who self-categorized as of Hispanic ethnicity, those born outside of the United States were separately coded due to different risks of maltreatment associated with immigrant status (Zhang et al., 2021). Maternal age at the time of birth was coded categorically (< 20 years, 20–24 years, 25–29 years, and 30+ years). Parity, or birth order, was categorized as first live birth, second or third birth, or fourth or higher birth. Birth payment method was used as a proxy for socioeconomic status and coded dichotomously (public insurance or private insurance, including self-pay). In California, uninsured women who present for delivery are retroactively enrolled in the state’s Medicaid program and therefore were coded as publicly insured (California Welfare and Institutions Code, 2022). Paternity was coded dichotomously as established or missing. Establishment of paternity was determined according to an anonymized indicator variable derived from the presence of a second parental name in the birth record. Because the California Health and Safety Code (2016) specifies that a second parent should not be listed in the birth record unless the parents are married or have signed a voluntary declaration of paternity at the time of delivery, this is a conservative estimate of paternal engagement. This convention of establishing paternity has been used in prior birth cohort analyses (Hutchins et al., 2022). Receipt of prenatal care was coded categorically based on the trimester during which the first recorded prenatal visit occurred (first, second, or third or no recorded prenatal care). Low birth weight was coded dichotomously (1 = <* 2500 g*, 0 = ≥ 2500 *g*).

### Statistical Analyses

Pearson’s chi-square tests and *t*-tests were used to examine frequencies of infants with and without documented PSE across covariates. Sankey diagrams were used to illustrate the proportion of children who transitioned from an investigation for alleged maltreatment to a foster care placement and mean days from birth to investigation to initial placement. The Sankey diagrams also show the child’s custody or placement status at age 3, although transitions in and out of foster care during the 3-year study period are not shown for ease of illustration and consistency with our focal outcome and models.

We estimated the likelihood a child was in nonparental custody at age 3 by specifying an adjusted multivariable generalized linear model, based on a Poisson distribution and a log link, with a robust standard error adjustment (Zou, 2004). Findings are presented as risk ratios (RR) with 95% confidence intervals (CI). This model specification is recommended to generate measures of association that are both interpretable and conservative in situations where odds ratios may overestimate group differences given the base rate of the outcome (Nurminen, 1995). All analyses were completed using Stata version 16.0.

## Results

### Birth Cohort Characteristics

Of the 22,855 infants who were the subject of a CPS investigation during their first year, 26.2% were identified during hotline screening as experiencing PSE. Variability across maternal race and ethnicity was observed, with higher prevalence of PSE among White mothers than Black, U.S.-born Hispanic, or foreign-born Hispanic mothers. Documented PSE was lower among infants born to mothers younger than 20 at time of birth and those who received early prenatal care and higher among those with public health insurance, without paternal information in the birth record, and who delivered infants with low birth weight. Birth characteristics along with pairwise chi-square tests of independence are included in Table 1.

**Table 1:** Sociodemographic characteristics and CPS involvement of births in California in 2017 Investigated for maltreatment in infancy by identified prenatal substance exposure

	Investigated	PSE	No PSE	PSE prevalence
	(*N* = 22,855)	(26.2%)	(73.8%)	
	*n*	Column %	Column %	Row %
Outcomes at age 3				
Ever in foster care	6560	48.6	21.7	44.3**
Adoption or guardianship	2081	21.8	4.6	62.5**
Nonparental custody	4219	34.9	12.6	49.5**
*Sociodemographic and pregnancy characteristics*				
Maternal race and ethnicity				
White	5691	33.4	21.9	35.1**
Black	3277	15.6	13.9	28.4*
Asian or Pacific Islander	855	2.6	4.2	18.1**
Native American or Alaska Native	316	2.1	1.1	38.9**
All other races	644	3.5	2.6	32.0*
U.S.-born Hispanic	9049	37.4	40.4	24.7**
Foreign-born Hispanic	3023	5.6	15.9	11.1**
Maternal age				
< 20	1646	3.4	8.5	12.5**
20–24	5710	23.1	25.6	24.2**
25–34	11,762	57.3	49.4	29.1**
≥ 35	3731	16.2	16.4	25.9
Parity				
First birth	6163	25.0	27.7	24.2**
Second or third birth	10,235	43.9	45.1	25.7
Fourth or higher birth	6401	30.6	27.1	28.5**
Birth payment method				
Public	18,269	84.8	78.2	27.7**
Private	4535	14.7	21.7	19.4
Paternity				
Established	16,581	58.6	77.5	21.1**
Missing	6274	41.4	22.5	39.4
Prenatal care				
First trimester	16,098	62.6	73.2	23.2**
Second trimester	4587	22.0	19.4	28.6**
Third trimester, none, or missing	2170	15.4	7.4	42.4**
Low birth weight				
< 2500g	2887	19.6	10.2	40.6**
≥ 2500g	19,968	80.4	89.9	24.1

Missing values: maternal age (*n* = 6, 0.03%), birth payment method (*n* = 51, 0.22%), parity (*n* = 56, 0.25%)

Pairwise comparison p-values: ** <0.001, * <0.01

In the first 3 years of life, more than 28% of all investigated infants experienced a foster care placement and 9.1% were adopted or had custody transferred through a guardianship placement. The share of investigated infants who experienced a foster care placement varied notably by PSE status. Among infants with documented PSE, 48.6% experienced a placement during the first 3 years, compared to 21.7% of those without PSE. By age 3, 21.8% of infants with documented PSE had experienced a transfer of custody via adoption or guardianship versus 4.6% of those without PSE. When including infants in an open foster care episode at age 3, 34.9% of infants with identified PSE were in nonparental custody, compared to 12.6% of those without identified PSE.

### Timing and Proportion of CPS Outcomes

**Fig. 1:**
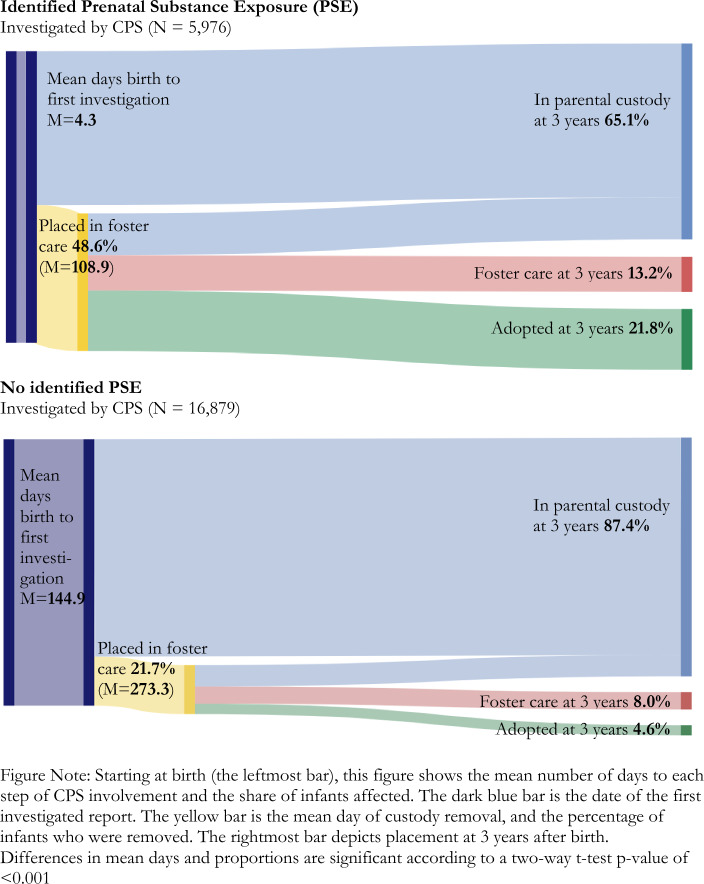
Timing and proportion of CPS involvement over 3 years among investigated infants with and without identified PSE

Figure 1 presents patterns of system involvement by PSE through a Sankey diagram. Starting at birth, we report the mean number of days to each step of CPS involvement and the share of a given group of investigated infants affected. This diagram illustrates an accelerated timeline of system penetration among PSE-identified infants. Mean days from birth to first investigated report differed significantly according to PSE status, with 4.3 days (*SD* = 28.6) on average between birth and first CPS report for those with identified PSE, compared to 144.9 days (*SD* = 113.0) for those without. Among the 48.6% of PSE-identified infants who experienced a foster care placement, the first removal occurred 108.9 days (*SD* = 226.9) after birth, on average, compared to 273.3 days (*SD* = 280.5) for infants without identified PSE. To assess whether this acceleration related primarily to the earlier date of first investigated report, we also inspected the significance of the difference between average days from first investigation to first foster care placement. This varied significantly, with a mean of 105.4 days (*SD* = 225.4) for PSE-identified infants, compared to 168.3 days (*SD* = 253.2) for those infants without PSE, a difference of more than 2 months. During the 3-year analytic period, PSE-identified infants spent an average of 380.1 days (*SD* = 472.7) in nonparental custody, compared to 122.9 days (*SD* = 291.0) for infants without identified PSE. Average days were compared using *t*-tests; all were found to differ significantly by PSE status (*p* < .001; statistics not shown).

### Regression Estimates

Table 2 shows results from unadjusted and adjusted generalized linear models, estimating the relative risk of being outside of the parental home 3 years after birth for all investigated infants. After controlling for maternal sociodemographic characteristics, infants with identified PSE were 2.2 times as likely to be out of the parental home at age 3 compared to those investigated by CPS without documented PSE. We tested for interactions to see if risk differed based on levels of birth and maternal covariates and our measure of PSE but did not find evidence of significant interactions among the included covariates (results not shown).

**Table 2:** Out-of-home placement at 3 years

	RR	95% CI
Unadjusted risk ratio (PSE vs. not)	2.76	2.62, 2.91**
Adjusted risk ratio (PSE vs. not)	2.16	2.05, 2.29**
*Covariates*		
Maternal race and ethnicity (ref. = non-Hispanic white)		
Non-Hispanic Black	0.94	0.87, 1.02
Asian and Pacific Islander	0.65	0.54, 0.79**
Native American or Alaska Native	1.12	0.92, 1.35
All other races	1.02	0.90, 1.17
U.S.-born Hispanic	0.90	0.84, 0.96*
Foreign-born Hispanic	0.54	0.48, 0.61**
Maternal age at birth (ref. = 25 − 34)		
< 20	1.28	1.13, 1.46**
20–24	1.21	1.13, 1.29**
≥ 35	0.89	0.83, 0.97*
Birth payment method (ref. = private)		
Public	1.36	1.25, 1.47**
Prenatal care (ref. = first trimester)		
Second trimester	1.09	1.02, 1.16
Third trimester, none, or missing	1.30	1.21, 1.41**
Parity (ref. = first birth)		
Second or third birth	1.33	1.23, 1.44**
Fourth or higher birth	1.90	1.75, 2.08**
Paternity (ref. = established)		
Missing	1.98	1.88, 2.09**
Low birth weight (ref. = ≥ 2500 g)		
< 2500 g	1.17	1.09, 1.26**

Maternal Hispanic ethnicity was coded across all racial groups

P-value **<0.001; *<0.01

## Discussion

Our study found that in a cohort of infants investigated for maltreatment by CPS, more than a quarter had documented PSE. Those with identified PSE were more likely to be removed from parental custody and less likely to be in the parental home at age 3. The increased likelihood of nonparental custody at age 3 was robust to adjustment for maternal and birth characteristics found to be associated with custody interruption. Although prior studies have demonstrated increased risk of maltreatment and custody interruption among infants with PSE compared to the general population (Prindle et al., 2018), our analysis of a subpopulation of investigated infants highlights the additional risk conferred by PSE status at the time of CPS intervention.

In 2019 in the United States, more than 80% of infants reported to CPS for PSE were screened in for an investigation or alternative response (Children’s Bureau, 2021). Our study found investigations occurred in roughly 97% of CPS reports with identified PSE in California. Given that this study occurred in a state with nonuniversal reporting for PSE—where mandated reporters are required to file an allegation of abuse and neglect only when current or on-going family risk is identified—it is likely that the PSE-identified infants in this sample are a highly vulnerable subgroup of all substance-exposed infants (Prindle et al., 2018). The high prevalence of investigatory responses may indicate that this discretionary reporting policy is effectively identifying high-risk families, although it is unknown whether the discretionary thresholds for reporting are consistently applied. In the context of shifting national child welfare policy, it is important that scholarship assess the circumstances of PSE identification and reporting and identify strategies to adopt a public health model of family preservation and treatment without compromising child safety (Lloyd Sieger et al., 2022; Madora et al., 2022).

One key finding that emerged was the young age of PSE infants at their first investigation (4.3 days old). This is expected because hospital providers are instructed to report infants born with PSE who have identified maltreatment risks prior to hospital discharge. In our sample of investigated infants, 98.6% of PSE-identified infants compared to 22.5% of infants without documented PSE were investigated in the first month of life, otherwise known as the neonatal period—a time of tremendous physical vulnerability. A recent longitudinal analysis found that infants reported to CPS as neonates were more likely to experience adoption (Magruder & Berrick, 2022). Our results confirm and extend this finding by adding the dimension of PSE, which is often a contributing factor for a neonatal CPS report (Lynch et al., 2018). To determine whether the additional risk among those with PSE was an artifact of younger age, we restricted our sample to infants reported in the first month of life and reran all analyses. We found that PSE-identified infants were still 30% more likely to be out of the parental home at age 3 in both crude [RR = 1.3; CI (1.2, 1.4)] and adjusted [RR = 1.3; CI (1.2, 1.3)] models, suggesting independent risk associated with PSE status.

While not the focus of our analysis, we did identify variation in the risks identified on the hotline screening tool between PSE and non-PSE infants. Among non-PSE infants, the most prevalent identified risk factor was exposure to domestic violence (32.8%), which was identified in only 0.47% of PSE infants. This is likely because CPS report data includes only what is known to and considered pertinent by the reporter at the time of the report. Variation in identified family risk attributes may result more from differences in reporter type and report timing rather than differences in family presentation.

We found that infants investigated for PSE had an accelerated pattern of subsequent system involvement, with PSE-identified infants being removed from the home quicker after an investigation compared to infants investigated for other reasons. Nearly 1 in 2 PSE-identified infants who were removed from the parental home were in a permanent adoptive or guardianship placement by age 3, compared to 21.4% of removed infants without documented PSE. This is an important finding given the dual objectives of CPS to support families and expedite permanency for children. The Adoption and Safe Families Act (1997) established permanency timelines to reduce extended stays in foster care and other temporary placements. Federal policy encourages states to establish permanency for children within 2 years of out-of-home placement. Scholars have argued that permanency and substance use disorder (SUD) recovery timelines are misaligned (Adlin Bosk et al., 2017). In a study showing the impact of SUD treatment on custody reunification, only 20% of the sample had been reunified within 2 years of treatment entry (Huang & Ryan, 2011). However, delaying permanent placements may not be advisable, because later age at removal among infants with PSE who are ultimately adopted from foster care is associated with worse developmental consequences (Tung et al., 2020).

In our study, nearly 5 times as many PSE infants were in permanent adoptive or guardianship placements at age 3 compared to infants involved with CPS for other reasons. This may speak to the complexity of perinatal SUD (Pentecost et al., 2021), the high prevalence of mental health complexities among women with SUD (Williams et al., 2021), and the association between parental substance use and child maltreatment (Palmer et al., 2022). Studies have shown that many women who achieve abstinence during pregnancy return to substance use within a year of giving birth (Forray et al., 2015). Additionally, as few as 15% of postpartum women with SUD receive any treatment (Faherty et al., 2021). Research has suggested that integrated service models, which provide support in areas of co-occurring concern, such as substance use, mental health, and housing, contribute more to reunification outcomes than child welfare interventions alone (Marsh et al., 2006; Bosk et al., 2019). Family-centered treatment, an evidence-based approach to preserving family cohesion, parental competence, and child safety and well-being (Wood et al., 2022), is a promising direction in the treatment and care of these vulnerable and complex-risk family units (Pierce et al., 2022). Integrated services may also be an effective strategy to respond to Federal legislation requiring service referrals for parents and children affected by PSE as a part of a comprehensive plan of safe care (Lloyd et al., 2019).

### Limitations

We recognize several limitations in our analyses. Given that PSE is differentially detected and reported, particularly for alcohol use (Chang, 2020), it is possible that infants reported to CPS for reasons other than PSE were misclassified on the hotline screening form.

Although we categorized custody outcomes at age 3, we did not model transitions that occurred within the 3-year period. As such, we cannot speak to whether 3-year custody outcomes occurred after failed reunification attempts. Similarly, our decision to look at 3-year outcomes did not capture custody transitions or interruptions in later childhood. Finally, because our analyses focused on California administrative records, although representing a large and diverse population, we cannot assume that our results are generalizable to other populations, particularly given the unique policy landscape in California guiding CPS referrals and responses to PSE.

## Conclusion

Given the unique risk profile of infants investigated by CPS with PSE, it is important to focus future research efforts on understanding the circumstances around CPS involvement in cases of PSE, particularly in settings where hotline and screening tool information can be used to augment case management system data. Additionally, studies can examine reunification attempts and service provision to assess the impact of CPS intervention efforts to maintain parental custody when possible. Finally, research should look at maternal history of CPS involvement and siblings to ascertain the impact of prior custody loss on subsequent CPS interactions.

In summary, our findings indicate that in a high-risk subgroup of infants with PSE, custody interruptions happen more often and quicker than among other CPS-involved infants. These findings could indicate that selective neonatal reporting is effective at identifying children most in need of CPS intervention. They also suggest the need for additional services tailored to families affected by substance use to optimize outcomes within current mandated permanency timelines.

## Data Availability

Data are available to research team under a data sharing agreement that prohibits re-release due to the nonpublic nature of the data.

## References

[CR1] Adlin Bosk E, Van Alst D, Van Scoyoc A (2017). A chronic problem: Competing paradigms for substance abuse in child welfare policy and practice and the need for new approaches. The British Journal of Social Work.

[CR2] Adoption and Safe Families Act, Pub. L. 105 – 89 (1997). https://www.congress.gov/105/plaws/publ89/PLAW-105publ89.pdf

[CR3] Austin AE, Gest C, Atkeson A, Berkoff MC, Puls HT, Shanahan ME (2021). Prenatal substance exposure and child maltreatment: A systematic review. Child Maltreatment.

[CR4] Benningfield MM, Arria AM, Kaltenbach K, Heil SH, Stine SM, Coyle MG, Fischer G, Jones HE, Martin PR (2010). Co-occurring psychiatric symptoms are associated with increased psychological, social, and medical impairment in opioid dependent pregnant women. The American Journal on Addictions.

[CR5] Bosk EA, Paris R, Hanson KE, Ruisard D, Suchman NE (2019). Innovations in child welfare interventions for caregivers with substance use disorders and their children. Children and Youth Services Review.

[CR6] California Department of Social Services (2021). *SDM policy and procedures manual*. https://www.cdss.ca.gov/Portals/9/Child-Welfare-Programs/Child-Welfare-Protection/SDM-Policy-Procedure-Manual-2021.pdf

[CR7] California Health and Safety Code, § 102425 (2016). https://leginfo.legislature.ca.gov/faces/codes_displaySectionxhtml?sectionNum=102425&lawCode=HSC

[CR8] California Penal Code, § 11165.13 (2000). https://leginfo.legislature.ca.gov/faces/codes_displaySection.xhtml?sectionNum=11165.13.&lawCode=PEN

[CR9] California Welfare and Institutions Code, § 14148 (2022). https://leginfo.legislature.ca.gov/faces/codes_displaySectionxhtml?sectionNum=14148&lawCode=WIC

[CR10] Center for Behavioral Health Statistics and Quality (2020). *2019 National Survey on Drug Use and Health*. https://www.samhsa.gov/data/release/2019-national-survey-drug-use-and-health-nsduh-releases

[CR11] Chang G (2020). Maternal substance use: Consequences, identification, and interventions. Alcohol Research: Current Reviews.

[CR12] Children’s Bureau (2019). *Child maltreatment 2017*. https://www.acf.hhs.gov/cb/report/child-maltreatment-2017

[CR13] Children’s Bureau (2020). *Child maltreatment 2018*. https://www.acf.hhs.gov/cb/report/child-maltreatment-2018

[CR14] Children’s Bureau (2021). *Child maltreatment 2019*. https://www.acf.hhs.gov/cb/report/child-maltreatment-2019

[CR15] Coleman-Cowger VH, Oga EA, Peters EN, Trocin KE, Koszowski B, Mark K (2019). Accuracy of three screening tools for prenatal substance use. Obstetrics and Gynecology.

[CR16] Crowley DM, Connell CM, Jones D, Donovan MW (2019). Considering the child welfare system burden from opioid misuse: Research priorities for estimating public costs. American Journal of Managed Care.

[CR17] Cuccaro-Alamin S, Foust R, Vaithianathan R, Putnam-Hornstein E (2017). Risk assessment and decision making in child protective services: Predictive risk modeling in context. Children and Youth Services Review.

[CR18] Ellsworth MA, Stevens TP, D’Angio CT (2010). Infant race affects application of clinical guidelines when screening for drugs of abuse in newborns. Pediatrics.

[CR19] Ettekal I, Eiden RD, Nickerson AB, Molnar DS, Schuetze P (2020). Developmental cascades to children’s conduct problems: The role of prenatal substance use, socioeconomic adversity, maternal depression and sensitivity, and children’s conscience. Development and Psychopathology.

[CR20] Faherty LJ, Heins S, Kranz AM, Stein BD (2021). Postpartum treatment for substance use disorder among mothers of infants with neonatal abstinence syndrome and prenatal substance exposure. Women’s Health Reports.

[CR21] Forray A, Merry B, Lin H, Ruger JP, Yonkers KA (2015). Perinatal substance use: A prospective evaluation of abstinence and relapse. Drug and Alcohol Dependence.

[CR22] Frame L (2002). Maltreatment reports and placement outcomes for infants and toddlers in out-of-home care. Infant Mental Health Journal.

[CR23] Ghertner R, Waters A, Radel L, Crouse G (2018). The role of substance use in child welfare caseloads. Children and Youth Services Review.

[CR24] Huang H, Ryan JP (2011). Trying to come home: Substance exposed infants, mothers, and family reunification. Children and Youth Services Review.

[CR25] Hutchins HJ, Harley KG, Wallenborn JT, Abrams B, Reno R, Parrish JW (2022). Breastfeeding duration and reported child maltreatment in a population-based alaskan birth cohort. Journal of Family Violence.

[CR26] Lewis MA, Giovannoni JM, Leake B (1997). Two-year placement outcomes of children removed at birth from drug-using and non-drug-using mothers in Los Angeles. Social Work Research.

[CR27] Lloyd MH (2018). Poverty and family reunification for mothers with substance use disorders in child welfare. Child Abuse Review.

[CR28] Lloyd MH, Luczak S, Lew S (2019). Planning for safe care or widening the net?: A review and analysis of 51 states’ CAPTA policies addressing substance-exposed infants. Children and Youth Services Review.

[CR29] Lloyd Sieger MH, Nichols C, Chasnoff IJ (2022). Child abuse Prevention and Treatment Act, family care plans and infants with prenatal substance exposure: Theoretical framework and directions for future research. Infant and Child Development.

[CR30] Lynch S, Sherman L, Snyder SM, Mattson M (2018). Trends in infants reported to child welfare with neonatal abstinence syndrome (NAS). Children and Youth Services Review.

[CR31] Madora M, Wetzler S, Jose A, Bernstein PS (2022). Pregnant and Postpartum People with Substance Use Disorders: Understanding the Obstetrical Care Provider’ s Roles and Responsibilities. Maternal and Child Health Journal.

[CR32] Magruder J, Berrick JD (2022). A longitudinal investigation of infants and out-of-home care. Journal of Public Child Welfare.

[CR33] Marsh JC, Ryan JP, Choi S, Testa MF (2006). Integrated services for families with multiple problems: Obstacles to family reunification. Children and Youth Services Review.

[CR34] Meinhofer A, Angleró-Díaz Y (2019). Trends in foster care entry among children removed from their homes because of parental drug use, 2000 to 2017. JAMA Pediatrics.

[CR35] Nurminen M (1995). To use or not to use the odds ratio in epidemiologic analyses?. European Journal of Epidemiology.

[CR36] Pallatino C, Chang JC, Krans EE (2021). The intersection of intimate partner violence and substance use among women with opioid use disorder. Substance Abuse.

[CR37] Palmer L, Font S, Eastman AL, Guo L, Putnam-Hornstein E (2022). What does child protective services investigate as neglect? A population-based study. Child Maltreatment.

[CR38] Paris R, Herriott AL, Maru M, Hacking SE, Sommer AR (2020). Secrecy versus disclosure: Women with substance use disorders share experiences in help seeking during pregnancy. Maternal and Child Health Journal.

[CR39] Patrick SW, Schiff DM, Committee on Substance Use and Prevention, Ryan, S. A., Quigley, J., Gonzalez, P. K., & Walker, L. R. (2017). A public health response to opioid use in pregnancy. Pediatrics.

[CR40] Pentecost R, Latendresse G, Smid M (2021). Scoping review of the associations between perinatal substance use and perinatal depression and anxiety. Journal of Obstetric Gynecologic & Neonatal Nursing.

[CR41] Pierce BJ, Muzzey FK, Bloomquist KR, Imburgia TM (2022). Effectiveness of family centered treatment on reunification and days in care: Propensity score matched sample from Indiana child welfare data. Children and Youth Services Review.

[CR42] Prindle JJ, Hammond I, Putnam-Hornstein E (2018). Prenatal substance exposure diagnosed at birth and infant involvement with child protective services. Child Abuse & Neglect.

[CR43] Putnam-Hornstein E, Ghaly M, Wilkening M (2020). Integrating data to advance research, operations, and client-centered services in California. Health Affairs.

[CR44] Putnam-Hornstein E, Needell B (2011). Predictors of child protective service contact between birth and age five: An examination of California’s 2002 birth cohort. Children and Youth Services Review.

[CR45] Putnam-Hornstein E, Prindle JJ, Leventhal JM (2016). Prenatal substance exposure and reporting of child maltreatment by race and ethnicity. Pediatrics.

[CR46] Putnam-Hornstein E, Simon JD, Eastman AL, Magruder J (2015). Risk of re-reporting among infants who remain at home following alleged maltreatment. Child Maltreatment.

[CR47] Racine N, McDonald S, Chaput K, Tough S, Madigan S (2021). Pathways from maternal adverse childhood experiences to substance use in pregnancy: Findings from the all our families cohort. Journal of Women’s Health.

[CR48] Radel, L., Baldwin, M., Crouse, G., Ghertner, R., & Waters, A. (2018). *Substance use, the opioid epidemic, and the child welfare system: Key findings from a mixed methods study*. https://aspe.hhs.gov/reports/substance-use-opioid-epidemic-child-welfare-system-key-findings-mixed-methods-study

[CR49] Rebbe R, Mienko JA, Brown E, Rowhani-Rahbar A (2019). Hospital variation in child protection reports of substance exposed infants. The Journal of Pediatrics.

[CR50] Schneiderman JU, Prindle J, Putnam-Hornstein E (2021). Infant deaths from medical causes after a maltreatment report. Pediatrics.

[CR51] Smith BD, Testa MF (2002). The risk of subsequent maltreatment allegations in families with substance-exposed infants. Child Abuse & Neglect.

[CR52] Smith DK, Johnson AB, Pears KC, Fisher PA, DeGarmo DS (2007). Child maltreatment and foster care: Unpacking the effects of prenatal and postnatal parental substance use. Child Maltreatment.

[CR53] Stritzel H (2022). State-level changes in health insurance coverage and parental substance use-associated foster care entry. Social Science & Medicine.

[CR54] Terplan M, Kennedy-Hendricks A, Chisolm MS (2015). Prenatal substance use: Exploring assumptions of maternal unfitness. Substance Abuse: Research and Treatment.

[CR55] Tung I, Christian-Brandt AS, Langley AK, Waterman JM (2020). Developmental outcomes of infants adopted from foster care: Predictive associations from perinatal and preplacement risk factors. Infancy.

[CR56] Vaithianathan R, Rouland B, Putnam-Hornstein E (2018). Injury and mortality among children identified as at high risk of maltreatment. Pediatrics.

[CR57] Velez ML, Montoya ID, Jansson LM, Walters V, Svikis D, Jones HE, Chilcoat H, Campbell J (2006). Exposure to violence among substance-dependent pregnant women and their children. Journal of Substance Abuse Treatment.

[CR58] Weber A, Miskle B, Lynch A, Arndt S, Acion L (2021). Substance use in pregnancy: Identifying stigma and improving care. Substance Abuse and Rehabilitation.

[CR59] Williams JR, Girdler S, Williams W, Cromeens MG (2021). The effects of co-occurring interpersonal Trauma and gender on opioid use and misuse. Journal of Interpersonal Violence.

[CR60] Wood, T., Fuller-Holden, J., Sullivan, M., McDuffie, J., & Glickman, S. (2022). *Family centered treatment: Cultivating hope through innovation*. https://static1.squarespace.com/static/57850a09d2b857904415f3fb/t/6220bc3b9f274b3f990b3b69/1646312512999/FCT+2022+White+Paper.pdf

[CR61] Wu SS, Ma CX, Carter RL, Ariet M, Feaver EA, Resnick MB, Roth J (2004). Risk factors for infant maltreatment: A population-based study. Child Abuse & Neglect.

[CR62] Zhang L, Bo A, Lu W (2021). To unfold the immigrant paradox: Maltreatment risk and mental health of racial-ethnic minority children. Frontiers in Public Health.

[CR63] Zou G (2004). A modified Poisson regression approach to prospective studies with binary data. American Journal of Epidemiology.

